# Understanding Heterogeneity in Individual Responses to Digital Lifestyle Intervention Through Self-Monitoring Adherence Trajectories in Adults With Overweight or Obesity: Secondary Analysis of a 6-Month Randomized Controlled Trial

**DOI:** 10.2196/53294

**Published:** 2024-03-20

**Authors:** Shiyu Li, Yan Du, Hongyu Miao, Kumar Sharma, Chengdong Li, Zenong Yin, Bradley Brimhall, Jing Wang

**Affiliations:** 1 Department of Kinesiology The Pennsylvania State University University Park, PA United States; 2 School of Nursing The University of Texas Health Science Center at San Antonio San Antonio, TX United States; 3 College of Nursing Florida State University Tallahassee, FL United States; 4 Center for Precision Medicine, Long School of Medicine The University of Texas Health Science Center at San Antonio San Antonio, TX United States; 5 Department of Public Health The University of Texas at San Antonio San Antonio, TX United States; 6 Department of Pathology and Laboratory Medicine, Long School of Medicine The University of Texas Health Science Center at San Antonio San Antonio, TX United States

**Keywords:** self-monitoring, adherence, weight loss, digital technology, behavior change, group-based trajectory modeling, precision health, mobile phone

## Abstract

**Background:**

Achieving clinically significant weight loss through lifestyle interventions for obesity management is challenging for most individuals. Improving intervention effectiveness involves early identification of intervention nonresponders and providing them with timely, tailored interventions. Early and frequent self-monitoring (SM) adherence predicts later weight loss success, making it a potential indicator for identifying nonresponders in the initial phase.

**Objective:**

This study aims to identify clinically meaningful participant subgroups based on longitudinal adherence to SM of diet, activity, and weight over 6 months as well as psychological predictors of participant subgroups from a self-determination theory (SDT) perspective.

**Methods:**

This was a secondary data analysis of a 6-month digital lifestyle intervention for adults with overweight or obesity. The participants were instructed to perform daily SM on 3 targets: diet, activity, and weight. Data from 50 participants (mean age: 53.0, SD 12.6 y) were analyzed. Group-based multitrajectory modeling was performed to identify subgroups with distinct trajectories of SM adherence across the 3 SM targets. Differences between subgroups were examined for changes in clinical outcomes (ie, body weight, hemoglobin A_1c_) and SDT constructs (ie, eating-related autonomous motivation and perceived competence for diet) over 6 months using linear mixed models.

**Results:**

Two distinct SM trajectory subgroups emerged: the *Lower SM group* (21/50, 42%), characterized by *all-around low and rapidly declining SM*, and the *Higher SM group* (29/50, 58%), characterized by *moderate and declining diet and weight SM with high activity SM*. Since week 2, participants in the Lower SM group exhibited significantly lower levels of diet (*P*=.003), activity (*P*=.002), and weight SM (*P*=.02) compared with the Higher SM group. In terms of clinical outcomes, the Higher SM group achieved a significant reduction in body weight (estimate: −6.06, SD 0.87 kg; *P*<.001) and hemoglobin A_1c_ (estimate: −0.38, SD 0.11%; *P*=.02), whereas the Lower SM group exhibited no improvements. For SDT constructs, both groups maintained high levels of autonomous motivation for over 6 months. However, the Lower SM group experienced a significant decline in perceived competence (*P*=.005) compared with the Higher SM group, which maintained a high level of perceived competence throughout the intervention (*P*=.09).

**Conclusions:**

The presence of the Lower SM group highlights the value of using longitudinal SM adherence trajectories as an intervention response indicator. Future adaptive trials should identify nonresponders within the initial 2 weeks based on their SM adherence and integrate intervention strategies to enhance perceived competence in diet to benefit nonresponders.

**Trial Registration:**

ClinicalTrials.gov NCT05071287; https://clinicaltrials.gov/study/NCT05071287

**International Registered Report Identifier (IRRID):**

RR2-10.1016/j.cct.2022.106845

## Introduction

### Background

The prevalence of obesity is increasing at an alarming rate in the United States, with approximately 42% of US adults aged ≥20 years considered obese [1]. An unhealthy lifestyle is a major risk factor for obesity in adults in the United States [2]. The United States Preventative Task Force recommends behavioral lifestyle interventions that include healthy diet and physical activity for weight management to overcome the burden of obesity [3]. However, conventional in-person lifestyle interventions have often reported reduced effectiveness for weight loss owing to poor attendance and adherence rates [4]. Therefore, it is necessary to improve the effectiveness of lifestyle interventions to address the obesity epidemic.

The surge of digital health technologies has resulted in significant progress in digital lifestyle interventions for obesity management [5]. The literature consistently reports the comparable efficacy of digital lifestyle interventions to in-person interventions in promoting short-term weight loss and improving health behaviors [6,7]. However, emerging evidence suggests that digital interventions may not be as effective as in-person interventions in maintaining weight loss and preventing weight regain [7]. It is arguable that the efficacy of current digital lifestyle interventions is hindered by a static, *one-size-fits-all* approach, in which all participants receive the same intervention without considering their personal characteristics, needs, capabilities, and preferences [8,9]. Adaptive interventions, characterized by repeated adjustment of intervention types or doses based on personal responses, represent a promising approach for optimizing digital lifestyle interventions for obesity management [10].

Developing adaptive interventions requires the early identification of participant subgroups that respond differently to the intervention. During an adaptive intervention, interventionists decide which intervention option is suited for those based on responses in intermediate tailoring variables. Intermediate tailoring variables may either lie on the causal pathway of the intervention (eg, intervention mediators) or be early predictors of long-term outcomes of interest (eg, improved clinical outcomes) [11]. Previous adaptive lifestyle interventions have used initial weight loss as an intermediate tailoring variable because of its predictive role in weight loss success, yielding mixed findings [9,11,12]. Self-monitoring (SM) adherence could be a more advantageous alternative in 2 ways. First, SM is a key component of the causal pathway of lifestyle interventions. The primary mechanism driving weight loss in lifestyle interventions is self-regulation, which involves individuals consistently monitoring their diet, physical activity, and weight. This ongoing SM enables them to reflect on and adjust their behaviors in accordance with their behavioral and weight loss goals [13,14]. Second, evidence suggests that SM is an important predictor of weight loss. This dual role of SM adherence, acting both as a predictor and causal factor for weight loss, potentially makes it a more impactful and actionable intermediate tailoring variable in adaptive lifestyle interventions for weight loss.

Investigating “how” to individualize interventions requires the identification of modifiable factors that can differentiate participants with different levels of intervention response. Self-determination theory (SDT) offers a unique theoretical perspective by focusing on the processes by which individuals initiate and maintain behaviors over time [15]. According to SDT, meaningful and lasting health behavior change requires that an individual is autonomously motivated for the change (autonomous motivation) and perceives themselves to be competent in making the change (perceived competence) [16]. Increasing autonomous motivation and perceived competence through intervention strategies has consistently been linked to successful changes in health behaviors [17-19]. However, studies have rarely examined whether lifestyle intervention participants differ in within-individual changes over time in terms of autonomous motivation and perceived competence. Notably, Webber et al [20] found that autonomous motivation levels at week 4, rather than baseline levels, predicted adherence to SM. Thus, comparing longitudinal changes in autonomous motivation and perceived competence among participants with different levels of intervention responses may provide valuable insights for developing effective adaptive interventions.

Contrary to the straightforward use of initial weight loss as an intermediate tailoring variable, employing SM adherence in this role requires a comprehensive consideration. In digital lifestyle interventions, participants are typically instructed to perform daily SM of diet, physical activity, and weight [21]. However, defining SM adherence is complicated by the presence of multiple SM targets and arbitrary selection of adherence thresholds for each [22]. This hinders the straightforward grouping of participants as responders or nonresponders based on a single SM adherence cut-off point, an approach frequently used in previous studies where initial weight loss was the intermediate tailoring variable [12]. Moreover, empirical evidence has consistently demonstrated a decline in SM adherence over time across various targets, underscoring its dynamic nature [21]. Consequently, a key challenge is to effectively distinguish participant responsiveness based on adherence to multiple SM adherence, considering its dynamic nature. Specifically, this study aimed to simultaneously capture the between-individual and within-individual changes in multiple SM adherence over time during a lifestyle intervention.

Group-based multitrajectory modeling (GBMM) is a promising approach for detecting both between-individual and within-individual variations in SM adherence [23]. GBMM assumes that (1) the population is composed of a finite number of homogenous subpopulations, each defined by its unique developmental trajectories across various indicators [24], and (2) significant heterogeneity exists in developmental trajectories among different population subgroups [24]. Applying the GBMM to longitudinal SM adherence data may uncover participant response subgroups that may not be detected by conventional demographic or clinical characteristics. For example, Yang et al [25] applied GBMM and identified 3 distinct participant subgroups based on their adherence to SM of physical activity, weight, and blood glucose during a digital lifestyle intervention in adults with type 2 diabetes [25]. Among these subgroups, a “low and waning” category emerged, characterized by consistently lower and declining adherence to weight and blood glucose SM. Moreover, this subgroup differed significantly in multiple baseline demographic characteristics and had higher baseline A_1c_ levels compared with the other 2 subgroups. However, this study did not associate SM trajectory subgroups with changes in clinical outcomes.

Another significant advantage of GBMM is its capacity to compare longitudinal SM adherence trajectories across participant response subgroups, allowing interventionists to pinpoint “when” participants began exhibiting varying response levels. This can inform the selection of a “Decision Point” for future adaptive interventions, an essential time point for assessing individual responsiveness, and for adapting intervention strategies [26]. Selecting decision points requires careful consideration of an individual’s dynamic state and the moments when meaningful changes occur in tailoring variables [27,28]. Previous research has often failed to justify predetermined decision points. For example, when using “initial weight loss” as the intermediate tailoring variable, several studies have used various time points as the “Decision Point,” with durations ranging from 3 to 7 weeks [9,11,12]. However, evidence suggests that the choice of decision point might indeed impact weight loss outcomes. Taken together, comparing SM adherence trajectories across participant subgroups identified by GBMM might aid in establishing evidence-based decision points.

### Objectives

Taken together, this study aimed to use GBMM to identify participant subgroups based on their longitudinal adherence patterns to SM of diet, physical activity, and weight over 6 months in a behavioral lifestyle intervention enhanced by digital SM of multiple behaviors. We also aimed to explore whether the intervention responses differed among the identified SM subgroups with respect to weight loss, glycemic control, and blood pressure. Finally, we explored whether SDT accounted for differences in SM trajectories.

## Methods

### Study Design and Participants

This was a secondary study based on a subset of data obtained from a pilot randomized controlled trial. Full details of the parent trial protocol have been published previously [29]. In brief, 60 participants (50 of whom were included in this secondary analysis) participated in a technology-assisted lifestyle intervention between June 2021 and October 2022 and were randomly assigned to receive either a ketogenic diet or a low-fat low-calorie diet.

The key inclusion criteria were (1) being 18 years of age or older, (2) being overweight or obese (BMI≥25 kg/m^2^), (3) with or without self-reported type 2 diabetes, (4) with or without evidence of chronic kidney disease, (5) being able to read and write in English, and (6) owning a smartphone. Key exclusion criteria included (1) being pregnant or breastfeeding, (2) under sodium-glucose cotransporter-2 treatment, (3) inability to walk without assistance, (4) triglyceride ≥500 mg/dL or low-density lipoprotein ≥129 mg/dL, (5) having severe psychiatric disorders or chronic conditions that may interfere with study procedures, (6) planning to leave the city for over 2 weeks within the intervention duration, (7) enrollment in other diet or weight loss programs, and (8) being unable or willing to consent.

Participants completed assessments of clinical and psychosocial outcomes at baseline and at 3 and 6 months of the parent trial. Participants were instructed to perform daily SM of diet, physical activity, and weight using the Fitbit food log (Fitbit Inc, United States), Fitbit Inspire 2 (Fitbit Inc), and Withing Body Scale (Withings Inc). All SM devices were provided by the study team.

The participants received a weekly personalized feedback message via email. These messages were tailored to each individual, focusing on their performance in SM and how well they adhered to diet and physical activity goals. If a participant did not provide any SM data for a month, a research assistant would reach out via phone to remind them about the importance of SM and assist them with any issues. In addition, individual web sessions with interventionists were scheduled at the start of the study and then at the 3- and 5-month marks. These meetings aimed to address and resolve any challenges participants faced in adhering to the study’s procedures, including SM.

In this secondary analysis, we excluded participants (n=5) who withdrew from the parental trial. This was necessary because adherence in clinical trials is defined as “the degree to which the behavior of trial participants corresponds to the intervention assigned to them” [30]. Those who withdrew from the study no longer received the intervention, including SM. We also excluded participants who did not finish setting up the SM devices (n=5) because we were unable to assess their SM behaviors.

The parent trial was approved by the Institutional Review Board of UT Health San Antonio. All participants provided written informed consent and received financial compensation.

### Measures

#### Adherence to SM

Adherence to SM of diet, physical activity, and weight were measured daily and operationalized as binary variables (adherence=1, nonadherence=0) based on whether a participant logged at least 800 kcal of total caloric intake (adherent: logged ≥800 kcal), at least 500 steps (adherent: logged ≥500 steps), and body weight (adherent: with a body weight value entry). In cases where participants did not provide SM data for a specific SM target on a given day, such instances were coded as *0*, reflecting nonadherence to the prescribed SM activities.

We chose an 800 kcal/d threshold for defining adherence to diet SM, as it was found to be the best predictor of weight loss among other diet SM metrics from food tracking apps [31]. In addition, this threshold was deemed to be a plausible daily caloric intake and has been commonly used in previous studies as a threshold for dietary SM [32]. We used 500 steps/d as the threshold for defining adherence to physical activity SM, as it is considered a realistic daily step count and has been commonly used as a threshold in previous studies [33].

#### Clinical Outcomes

All measurements were collected in a fasting state by trained staff members using standardized protocols at the UT Health San Antonio School of Nursing at baseline and at 3 and 6 months. Weight (in kg) was measured using the mean of two measurements by a professional-grade digital scale, while participants wore light clothing with bare feet on the scale’s footpads. Systolic blood pressure (SBP) and diastolic blood pressure (DBP) were recorded as the average of 2 measurements using an Omron professional blood pressure machine (OMRON Group, Japan) while participants were in a relaxed sitting position. Fasting blood samples were stored at −80 °C. Hemoglobin A_1c_ (HbA_1c_) levels were analyzed using Quest Diagnostics (Dallas, TX).

#### Autonomous Motivation

Autonomous motivation for diet was assessed using the *autonomous regulation* subscale from the Treatment Self-Regulation Questionnaire for Diet [34]. The questionnaire includes various statements that represent different types of motivation for following a healthy diet based on SDT. Participants expressed their level of agreement with each statement using a 7-point Likert scale, where a score of 1 indicated “not at all true” and a score of 7 signified “very true.” Our analysis focused on 6 statements that measured autonomous motivation. The autonomous motivation score at each time point was determined by calculating the average score of the 6 items related to autonomous motivation. Cronbach α for autonomous motivation was 0.86 at baseline, 0.92 at 3 months, and 0.91 at 6 months, respectively.

#### Perceived Competence

Perceived competence in diet was assessed using the Perceived Competence Scale for Diet [35]. The scale presented participants with 4 statements assessing their belief in their ability to maintain a healthy diet. Participants rated their agreement with each statement on a 7-point Likert scale ranging from 1 (not at all true) to 7 (very true). The total perceived competence score was calculated as the average of the 4 items at each time point. Cronbach α for perceived competence for diet were 0.94 at baseline, 0.97 at 3 months, and 0.95 at 6 months, respectively.

#### Demographic Characteristics

Participant demographics were obtained from self-reported questionnaires and surveys at baseline. The demographic variables included age (years), sex, race and ethnicity, education, and annual household income.

### Statistical Analysis

#### Identifying Distinct Trajectories of Adherence to SM of Diet, Activity, and Weight

##### Overview

We established 3 daily binary variables to measure *adherence* versus *nonadherence* to SM of diet, activity, and weight, respectively. We then calculated the number of days of adherence to each SM target at 2-week intervals over 6 months.

We used the STATA PROC TRAJ package to fit GBMM using censored regressions as the link function to model the longitudinal trajectories of adherence to SM of diet, activity, and weight. The purpose of GBMM was to (1) identify the number of SM trajectory subgroups and (2) describe how these subgroups are distributed across populations.

##### Model Fitting

GBMM models were estimated using a 2-step approach as described in the study by Nagin et al [23]. In step 1, we estimated models with 2 to 5 subgroups for each of the SM targets (diet, physical activity, weight) separately with trajectories modeled as linear or quadratic functions of time within each model. The objective was to identify the types of distinctive trajectories that were important for representation in the GBMM model. In this step, we observed that the trajectories generally followed a linear time function, as evidenced by the significance of all the linear terms (Multimedia Appendix 1 [23]).

In step 2, we proceeded to estimate SM for all 3 SM targets together, with a linear time function and considering 2-4 subgroups. Our findings indicate that all estimated GBMM models satisfied our predefined criteria for model selection. Notably, the models with a greater number of subgroups demonstrated improved Bayesian Information Criterion scores.

After a closer examination of SM adherence trajectories for 2-4 subgroups, we observed that the diet SM adherence trajectories in models with 3 and 4 groups did not show significant differences compared with the 2-group model. In addition, the 2-group model effectively captured the distinct trajectories for each SM target, as revealed in step 1 of the analysis. Thus, our final preferred model for multitrajectory analysis consisted of 2 distinct groups for simplicity and ease of interpretation in subsequent multivariate analyses.

##### Model Selection

Given that the Bayesian Information Criteria and Bayes factor increased with the increasing number of subgroups, we selected the final GBMM model based on the following criteria: (1) model value of *P*≤.05, (2) trajectory shape similarity, (3) average posterior probability ≥0.7, (4) odds of correct classification ≥5.0, (5) minimum sample size for each subgroup ≥10%, (5) the proportion of a sample assigned to a certain group is close to the proportion estimated from the model, and (6) 99% CIs of the estimated proportion are reasonably narrow. Thus, a 2-group model with linear terms was considered to be the optimal model, and both groups had posterior probabilities of 0.97 or higher. The details of the model selection procedures are described in the Multimedia Appendix 1.

#### Examining Differences between SM Trajectory Subgroups on SM Adherence, Metabolic Outcomes, and Motivation

Continuous variables were first checked for normality using the Shapiro-Wilk test. To examine differences between SM trajectory subgroups on baseline characteristics, we performed an independent *t* test or Mann-Whitney *U* test for continuous variables, depending on data normality, and a chi-square test for categorical variables.

Linear mixed models (LMMs) were used to examine the differences between SM trajectory subgroups on SM adherence over time, changes in metabolic outcomes, changes in eating-related autonomous motivation, and changes in perceived competence for diet.

All LMMs included a random intercept, accounting for an individual’s deviation from the ground mean. Fixed effects for the LMMs included time (2-week intervals for SM adherence; baseline, 3 months, and 6 months for clinical outcomes and SDT constructs), allocated SM trajectory subgroup (Lower SM vs Higher SM), and interaction between time and SM trajectory subgroup. Given the primary focus of the parent trial on dietary interventions, we have consistently included diet type (ketogenic diet vs low-fat low-calorie diet) as a fixed effect in all LMMs to control for the potential influences of diet type on specific outcomes.

Considering the small sample size in this study, confounders such as age, sex, Hispanic ethnicity, annual household income, and education level were included in the full model and then selected using backward stepwise variable selection to identify the parsimonious model structure with insignificant (*P*>.05) confounders gradually removed. All statistical and data visualization procedures were performed using R version 4.2.1.

### Ethical Considerations

All study procedures were approved by the Institutional Review Board of The University of Texas Health Science Center at San Antonio (#20190528HU).

## Results

### Baseline Characteristics of Study Participants by SM Trajectory Groups

Two SM trajectory subgroups emerged: the *Lower SM group* (21/50, 42%), characterized by *all-around low and rapidly declining SM*, and the *Higher SM group* (29/50, 58%), characterized by *moderate and declining diet and weight SM with high activity SM.*

The baseline characteristics of the SM trajectory groups are presented in Table 1. Participants were middle-aged (age: 53.0 SD 2.6 y), mostly of Hispanic or Latino origin (28/50, 56%), and at a generally higher socioeconomic status and education level. We found that the 2 SM trajectory groups were significantly different at baseline; those in the Higher SM group were older compared with those in the Lower SM group. We also found that participants in the Higher SM group had higher baseline SBP and DBP levels.

**Table 1:** Baseline characteristics by self-monitoring (SM) trajectory groups.

		Total (n=50)	Lower SM (n=21)	Higher SM (n=29)	Between-group *P* value	
Age (years), mean (SD)		53. 0 (12.6)	47.1 (13.9)	57.2 (9.8)	.004	
Female, n (%)		32 (64)	16 (76.2)	16 (55.2)	.13	
Hispanic or Latino, n (%)		28 (56)	13 (61.9)	15 (51.7)	.08	
Type 2 diabetes, n (%)		25 (50)	8 (38.1)	17 (58.6)	.15	
Chronic kidney disease, n (%)		17 (34)	7 (33.3)	10 (34.5)	.93	
**Education, n (%)**						.86
	High school	6 (12)	3 (14.3)	3 (10.3)		
	Some college	19 (38)	7 (33.3)	12 (41.4)		
	College degree	13 (26)	5 (23.8)	8 (27.6)		
	Graduate degree	12 (24)	6 (28.6)	6 (20.7)		
**Annual household income (US $), n (%)**						.77
	<40,000	10 (20)	4 (19.1)	6 (20.7)		
	40,000-79,999	14 (28)	5 (23.8)	9 (31)		
	≥80,000	26 (52)	12 (57.1)	14 (48.3)		
**Treatment group, n (%)**						.54
	Ketogenic diet	26 (52)	12 (57.1)	14 (48.3)		
	Low-fat low-calorie diet	24 (48)	9 (42.9)	15 (51.7)		
Body weight (kg), mean (SD)		102.5 (22.1)	103.2 (26.2)	102.0 (19.1)	.86	
HbA_1c_^a,b^, mean (SD)		6.1 (1.1)	5.9 (1.2)	6.2 (1.1)	.47	
SBP^a,c^ (mm Hg), mean (SD)		132.0 (13.7)	126.0 (10.8)	136.4 (14.0)	.007	
DBP^d^ (mm Hg), mean (SD)		72.0 (9.2)	68.8 (9. 6)	74.3 (8.3)	.04	
Autonomous motivation^a^, mean (SD)		6.0 (0.8)	6.1 (0.9)	6.0 (0.8)	.75	
Perceived competence, mean (SD)		5.4 (1.0)	5.7 (0.9)	5.3 (1.0)	.29	

^a^Nonparametric Mann-Whitney *U* test was performed.

^b^HbA_1c_: hemoglobin A_1c_.

^c^SBP: systolic blood pressure.

^d^DBP: diastolic blood pressure.

### Adherence to Multiple SM by SM Trajectory Groups

**Figure 1:**
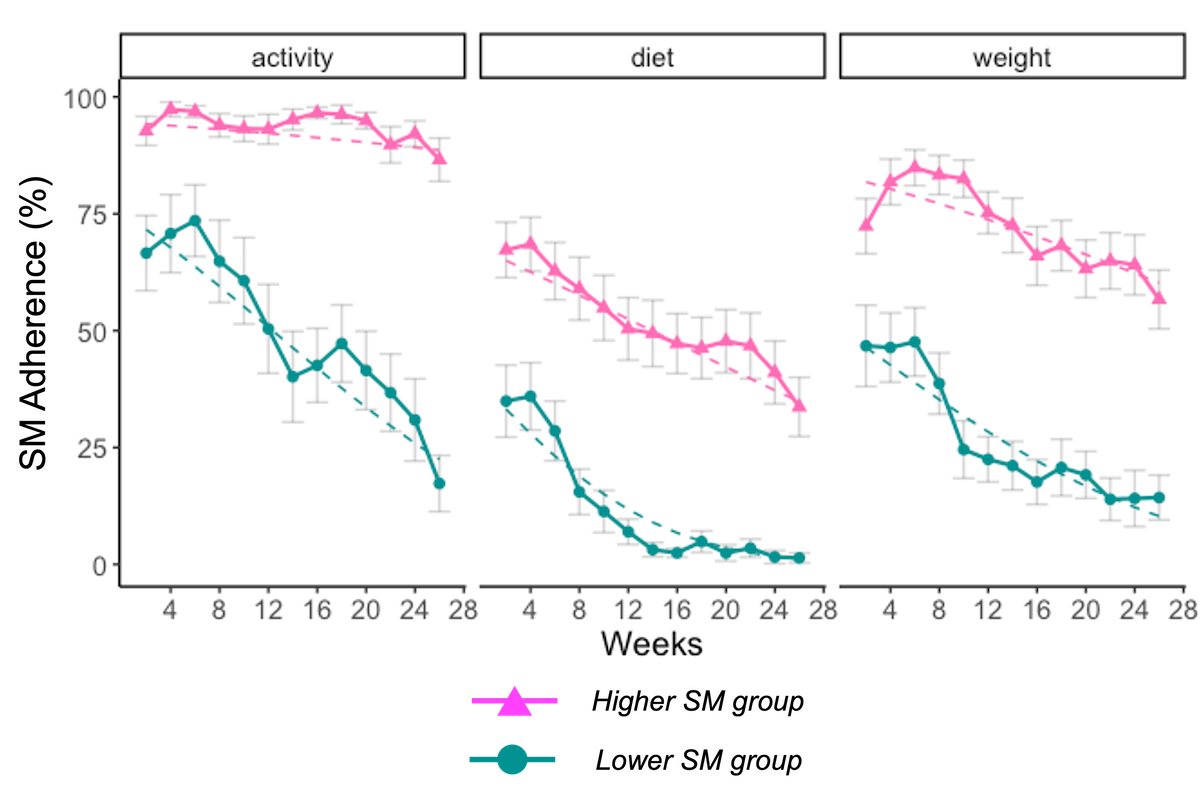
Adherence to multiple self-monitoring (SM) by trajectory subgroup.

Figure 1 presents the adherence to multiple SM by SM trajectory group. Overall, participants in the Lower SM group (circle) subgroup exhibited a low-to-moderate level of adherence to diet and weight SM. Levels of physical activity SM were slightly higher than diet and weight SM at the beginning of the intervention but decreased rapidly after approximately 1 month of intervention. The Higher SM group (triangle) exhibited a moderate level of diet and weight SM, whereas physical activity SM remained high and stable over 6 months.

At week 2, participants in the Lower SM group started having a significantly lower SM adherence for diet (mean difference: 30.36, SD 9.56%; *P*=.003), physical activity (mean difference: 25.26, SD7.73%; *P*=.002), and weight (mean difference: 23.96, SD 10.1%; *P*=.02) compared with the Higher SM group. The adjusted LMM further revealed a significant main effect of time and SM trajectory group on diet SM (estimate: −2.68, SD 0.2, *P*<.001), activity SM (estimate: −2.24, SD 0.21, *P*<.001), and weight SM (estimate: −2.68, SD 0.2, *P*<.001). In addition, there was a significant time and SM trajectory group interaction on activity SM (estimate: −1.85, SD 0.21, *P*<.001) and weight SM (estimate: −0.62, SD 0.22, *P*=.005), suggesting that participants in the Lower SM group decreased their adherence to activity and weight SM at a faster rate.

### Changes in Clinical Outcomes Between SM Trajectory Groups

**Figure 2:**
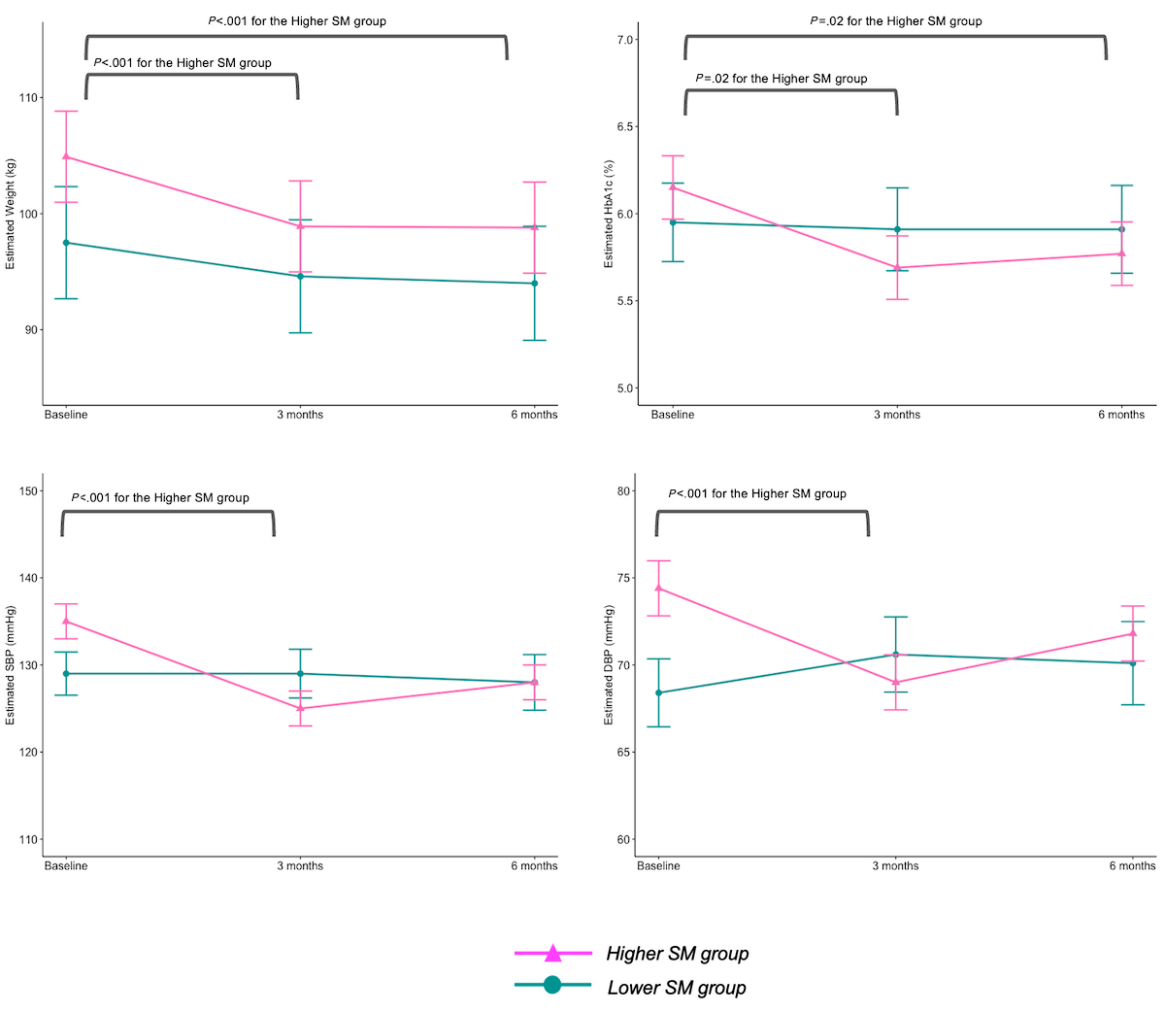
Estimated mean changes in clinical outcomes over time by self-monitoring (SM) trajectory group. DBP: diastolic blood pressure; HbA_1c_: hemoglobin A_1c_; SBP: systolic blood pressure.

Table 2 shows estimated mean changes in clinical outcomes over 6 months. Adjusted LMMs revealed significant interaction between time and SM trajectory group on weight, HbA_1c_, SBP, and DBP over 6 months. Specifically, participants in the Higher SM group significantly reduced body weight (estimate: −5.98, SE 0.87 kg, *P*<.001), HbA_1c_ (estimate: −0.46, SE 0.11%, *P*=.002), SBP (estimate: −9.71, SE 2.25 mmHg, *P*<.001), and DBP (estimate: −5.39, SE 1.49 mmHg, *P*=.008) from baseline to 3 months (Table 2, Figure 2). In addition, they maintained significant weight loss (estimate: −6.06, SE 0.87 kg, *P*<.001) and HbA_1c_ control at 6 months (estimate: −0.38, SE 0.11%; *P*=.02).

**Table 2:** Estimate mean changes and SE in clinical outcomes by self-monitoring (SM) trajectory group from baseline to 3 and 6 months.

	Baseline to 3 months				Baseline to 6 months		
	Lower SM group	Higher SM group	Between-group *P* value	Lower SM group		Higher SM group	Between-group *P* value
Weight (kg), mean (SE)	−2.86 (1.23)	−5.98 (0.87)^a^	.02	−3.48 (1.4)		−6.06 (0.87)^a^	.20
HbA_1c_ (%), mean (SE)	−0.04 (0.16)	−0.46 (0.11)^b^	.02	−0.03 (0.18)		−0.38 (0.11)^c^	.03
SBP^d^ (mm Hg), mean (SE)	−0.01 (3.05)	−9.71 (2.26)^e^	.048	−0.42 (3.42)		−6.44 (2.26)	.23
DBP^f^ (mm Hg), mean (SE)	2.15 (2.03)	−5.39 (1.49)^b^	.04	1.69 (2.29)		−2.59 (1.49)	.08

^a^Within-group: *P*<.001.

^b^Within-group: *P*<.01.

^c^Within-group: *P*<.05.

^d^SBP: systolic blood pressure.

^e^Within-group: *P*<.001.

^f^DBP: diastolic blood pressure.

### Changes in Autonomous Motivation and Perceived Competence by SM Trajectory Groups

**Figure 3:**
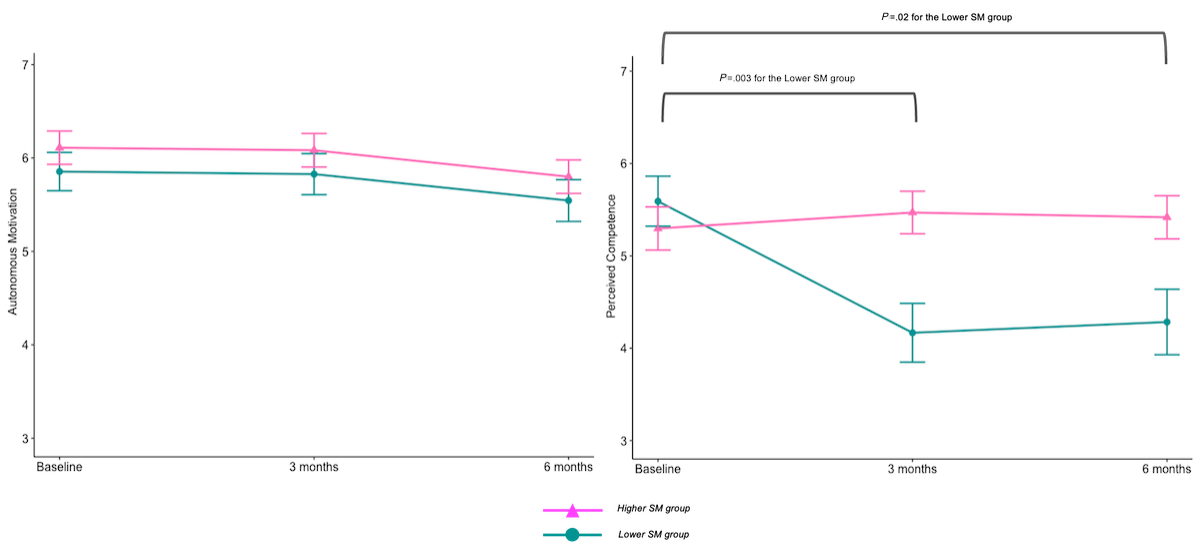
Estimated mean changes in autonomous motivation (left) and perceived competence (right) for diet over 6 months. SM: self-monitoring.

Longitudinal changes in autonomous motivation and perceived competence in diet are shown in Figure 3. Findings from the LMM showed that both SM trajectory groups maintained a high level of autonomous motivation for over 6 months.

The LMM reported a significant interaction effect of the SM trajectory group and time (*P*<.001), as well as a main effect for the SM trajectory group (Lower SM group vs Higher SM group). Posthoc analyses revealed that although the responders maintained a high level of perceived competence for diet throughout the intervention period, nonresponders experienced a significant decrease in perceived competence from baseline to 3 months (estimate: –1.42, SE 0.37, *P*=.003) and 6 months (estimate: −1.31, SE 0.4, *P*=.02). Furthermore, the Higher SM group showed a significantly higher level of perceived competence in diet at 3 months than the Lower SM group (*P*=.02).

### Attrition and Individual Session Attendance by SM Trajectory Groups

Table 3 presents the differences in attrition rates between subgroups at the 3- and 6-month data collection points as well as differences in attendance at individual sessions during months 3 and 5. Notably, no attrition was observed in the Higher SM group at 3 or 6 months. In addition, this group exhibited approximately 90% attendance in individual sessions.

**Table 3:** Attrition rate and individual session attendance by self-monitoring (SM) trajectory subgroup.

			Lower SM group, n (%)		Higher SM group, n (%)		Total, n (%)		*P* value
**Attrition**									
	3 months	6 (28.6)		0 (0)		6 (12)		.002	
	6 months	9 (42.9)		0 (0)		9 (18)		<.001	
**Individual session attendance**									
	3 months	14 (66.7)		27 (93.1)		41 (82)		.02	
	5 months	10 (47.6)		26 (89.7)		36 (72)		<.001	

## Discussion

### Principal Findings

In this study, we used GBMM and identified 2 participant subgroups exhibiting distinct SM trajectories during a 6-month lifestyle intervention in adults with overweight or obesity. Notably, we observed that the Lower SM group started displaying early low and rapidly declining levels of SM within the first 2 weeks. At 6 months, the Lower SM group did not show significant improvement in clinical outcomes and experienced a significant decline in perceived competence for diet compared with the Higher SM group. Our findings suggest that adherence to multiple SM may be a salient intermediate tailoring variable for identifying clinically meaningful participant subgroups. Moreover, the presence of the Lower SM group highlights the importance of early intervention, particularly within the initial 2 weeks, to positively impact clinical health outcomes. In addition, our study implies the potential of adopting early intervention strategies aimed at enhancing perceived competence in diet to benefit participants at risk of not responding well to digital lifestyle interventions.

### Comparison With Prior Work

Similar to previous studies, we found that older adults were more likely to be in the Higher SM group [25,36]. Consistent evidence suggests that age is among the most prominent predictors of adherence to behavioral lifestyle interventions for weight loss [4]. Older adults are willing to learn digital health technologies. When they have an internal desire to make lifestyle changes, they are likely to engage and explore various digital health technologies [37]. Thus, tailored intervention strategies are needed to improve adherence to digital SM among younger middle-aged participants [38].

Participants in the Lower SM group consistently exhibited lower levels of multitarget SM, even during the initial 2 weeks, and did not experience significant health benefits at 6 months. Frequent SM adherence during the early intervention phase is an important predictor of later weight loss [39]. Therefore, early SM is important for successful weight loss. However, individuals’ attitudes toward SM can change rapidly over a few days. As reported by Fausset et al [40] all participants initially exhibited strong motivation and a positive attitude toward SM activity, but after 2 weeks, a significant proportion experienced a shift in attitude, perceiving SM as ineffective, and subsequently discontinued its use. In line with previous research, our study highlights the first 2 weeks of the intervention as a critical time window for interventionists to identify *nonresponsive* individuals and provide tailored strategies. To optimize interventions and promote better long-term health outcomes, future studies should investigate the feasibility and effectiveness of providing additional support during this initial phase for at-risk individuals, and compare it with conventional *one-size-fits-all* interventions.

We observed that although SM subgroups displayed differing levels of SM adherence and health improvement, they also showed similarly high levels of autonomous motivation. This pattern highlights an intention-behavior gap in health behavior change, aligning with the Health Action Process Approach theory [41]. Health Action Process Approach conceptualizes behavior change as a process with 2 distinct phases: the motivational phase, where intentions are formed, and the volitional phase, where these intentions are put into action and maintained [41]. The intention-behavior gap emerges when there is a failure to translate intentions into actions. The consistently high levels of autonomous motivation observed in both subgroups indicate that participants were intending for health behavior change. However, the disparity in actual adherence and health improvements, particularly in the Lower SM group, suggests a lack of self-regulatory skills or strategies to actualize these intentions into sustainable action [42]. This finding highlights the necessity of identifying the specific phase of behavioral change that an individual is in when designing and implementing adaptive interventions. Accordingly, it is crucial that these interventions are adapted to either enhance motivation or facilitate the translation of intentions into health behaviors.

Although both SM trajectory groups initially had similar levels of motivation and perceived competence in diet, individuals in the Lower SM group experienced a significant decline in perceived competence in diet over time. This highlights the potential role of SM in maintaining perceived competence in a healthy lifestyle and sustaining long-term health behaviors. For the Higher SM group, regular SM and ongoing assessment of progress toward behavioral and weight loss goals could assist participants in gaining mastery in making lifestyle behavioral adjustments to achieve goals, which could explain the higher level of perceived competence in this group. Perceived competence has been identified as an effective target for achieving long-term health behavior changes [19]. A previous study on adults with type 2 diabetes found that targeting perceived competence in a short period (3 days) can lead to significant improvements in physical activity and glycemic control at 3 months [43]. Therefore, intervention strategies focusing on enhancing perceived competence for a healthy diet among low-SM adherents during the early phase of the intervention may be effective for long-term health outcomes.

Participants in the Higher SM group achieved clinically significant weight loss (≥5%) at 6 months, despite only monitoring their diet and weight at a moderate level within the initial 2 weeks of the intervention. This aligns with findings from Xu et al [36] and others that a moderate level of SM diet is associated with clinically significant weight loss. Expanding on their research, our study further demonstrated that a moderate level of both diet and weight SM during the early phase of intervention could be associated with significant health improvements. In contrast, we observed that participants in the Higher SM group not only demonstrated better adherence to SM but also higher engagement in study procedures other than SM and had no attrition. Given that attendance was also a significant predictor of weight loss [44], it becomes clear that future research should focus on disentangling the complex interplay between SM, intervention engagement, and health outcomes.

Previous studies have often associated reduced SM adherence with a failure of behavioral self-regulation and have focused on developing strategies to enhance SM adherence [29,45]. However, our study provides a different perspective, revealing that participants in the higher SM group achieved continued weight loss and maintained a high level of autonomous motivation and perceived competence in their diet, despite experiencing a decline in SM adherence between 3 and 6 months. This indicates the possibility of habit formation for healthy lifestyles and a voluntary *happy abandonment* of SM within the higher SM group. Similarly, Attig and Franke [46] reported that individuals might stop using activity SM devices once they have formed habitual exercise behaviors [46]. To optimize interventions, researchers must recognize that the role of SM lies in supporting behavioral self-regulation and sustaining motivation for a healthy lifestyle. If participants have established long-lasting motivation and formed habits for a healthy lifestyle, additional SM strategies may be unnecessary and wasteful of resources. Taken together, to optimize the SM component in lifestyle interventions, future studies need to use intensive longitudinal data collection and analytic strategies (ie, ecological momentary assessments) to unfold the dynamics between motivation for SM, motivation for a healthy lifestyle, and real-time lifestyle behavior change [47].

### Limitations

This study had several limitations. First, we focused on SM of diet, activity, and weight, as they were predominantly recommended for obesity management; however, adults living with overweight or obesity with comorbidities might also perform other types of SM (ie, blood glucose, blood pressure, etc), which could impact their changes in metabolic health during the intervention.

Second, although we defined SM adherence based on the current literature, the definitions are still arbitrary. Future studies are needed to examine the impact of various SM adherence criteria on the identification of SM trajectory subgroups to ensure replicability of our findings.

Third, the GBMM approach assigned participants to subgroups based on their closest SM trajectory for each target, which could obscure within-group variability in their SM trajectories for each target.

Fourth, owing to the small sample size, we had limited statistical power to identify additional SM trajectory subgroups.

Fifth, we assigned 0s to indicate nonadherence on days when participants did not provide SM data for a specific SM target. However, this approach could not differentiate between participants who completely withdrew from all the study procedures and those who merely neglected SM while adhering to other aspects of the study. These participants could appear indistinguishable when solely analyzing the SM adherence data. It is important to note that different strategies may be required for tailoring interventions. Therefore, future research should examine SM trajectory patterns more closely to explore methods that can effectively differentiate these participants.

Sixth, we chose 2 group model as the final GBMM model to balance the sample size of each subgroup and increase the power of the comparison between participant subgroups. However, there could have been unexplored SM trajectories in this study. Thus, future studies with larger sample sizes are warranted.

Seventh, the participants in our study were mostly from higher socioeconomic backgrounds with higher levels of education, which could limit the generalizability of our findings to a broader population. Furthermore, we excluded participants who did not finish setting up SM technologies or who withdrew from the parent trial. However, these participants might represent a distinct subgroup that requires different intervention strategies to improve their lifestyle and metabolic health.

Finally, the relatively short duration of our intervention limits our ability to thoroughly examine the long-term effects of different SM trajectory groups on sustaining health improvements.

### Conclusions

By modeling the longitudinal SM adherence trajectories using the GBMM, we identified distinct participant subgroups exhibiting varied responses to a digital lifestyle intervention in terms of SM adherence, clinical outcomes, and motivation factors. Our findings support the use of SM adherence as a salient proximal outcome for the early identification of participants at risk for *nonresponding*. Moreover, our research highlights the potential of effective intervention strategies aimed at improving perceived competence in the diet. The findings of our study hold the promise of informing the development of future adaptive lifestyle interventions for obesity management.

## Appendix

Multimedia Appendix 1 Group-based multitrajectory model procedures.

## Abbreviations

DBP: diastolic blood pressure
GBMM: group-based multitrajectory modeling
HbA_1c_: hemoglobin A_1c_
LMM: linear mixed model
SBP: systolic blood pressure
SDT: self-determination theory
SM: self-monitoring

## References

[1] Stierman B, Afful J, Carroll MD, Chen TC, Davy O, Fink S, Fryar CD, Gu Q, Hales CM, Hughes JP, Ostchega Y, Storandt RJ, Akinbami LJ (2021). National Health and Nutrition Examination Survey 2017–March 2020 prepandemic data files development of files and prevalence estimates for selected health outcomes. Centers for Disease Control and Prevention.

[2] Hruby A, Manson JE, Qi L, Malik VS, Rimm EB, Sun Q, Willett WC, Hu FB (2016). Determinants and consequences of obesity. Am J Public Health.

[3] Curry SJ, Krist AH, Owens DK, Barry MJ, Caughey AB, Davidson KW, Doubeni CA, Epling JW, Grossman DC, Kemper AR, Kubik M, Landefeld CS, Mangione CM, Phipps MG, Silverstein M, Simon MA, Tseng C, Wong JB, US Preventive Services Task Force (2018). Behavioral weight loss interventions to prevent obesity-related morbidity and mortality in adults: US preventive services task force recommendation statement. JAMA.

[4] Burgess E, Hassmén P, Pumpa KL (2017). Determinants of adherence to lifestyle intervention in adults with obesity: a systematic review. Clin Obes.

[5] Bhardwaj NN, Wodajo B, Gochipathala K, Paul DP 3rd, Coustasse A (2017). Can mHealth revolutionize the way we manage adult obesity?. Perspect Health Inf Manag.

[6] Mamalaki E, Poulimeneas D, Tsiampalis T, Kouvari M, Karipidou M, Bathrellou E, Collins CE, Panagiotakos DB, Yannakoulia M (2022). The effectiveness of technology-based interventions for weight loss maintenance: a systematic review of randomized controlled trials with meta-analysis. Obes Rev.

[7] Beleigoli AM, Andrade AQ, Cançado AG, Paulo MN, Diniz MD, Ribeiro AL (2019). Web-based digital health interventions for weight loss and lifestyle habit changes in overweight and obese adults: systematic review and meta-analysis. J Med Internet Res.

[8] König LM, Attig C, Franke T, Renner B (2021). Barriers to and facilitators for using nutrition apps: systematic review and conceptual framework. JMIR Mhealth Uhealth.

[9] Miller CK (2019). Adaptive intervention designs to promote behavioral change in adults: what is the evidence?. Curr Diab Rep.

[10] Lei H, Nahum-Shani I, Lynch K, Oslin D, Murphy SA (2012). A "SMART" design for building individualized treatment sequences. Annu Rev Clin Psychol.

[11] Almirall D, Nahum-Shani I, Sherwood NE, Murphy SA (2014). Introduction to SMART designs for the development of adaptive interventions: with application to weight loss research. Transl Behav Med.

[12] Sherwood NE, Butryn ML, Forman EM, Almirall D, Seburg EM, Lauren Crain A, Kunin-Batson AS, Hayes MG, Levy RL, Jeffery RW (2016). The BestFIT trial: a SMART approach to developing individualized weight loss treatments. Contemp Clin Trials.

[13] Kanfer FH (1970). Self-monitoring: Methodological limitations and clinical applications. J Consult Clin Psychol.

[14] Burke LE, Wang J, Sevick MA (2011). Self-monitoring in weight loss: a systematic review of the literature. J Am Diet Assoc.

[15] Ryan RM, Patrick H, Deci EL, Williams GC (2008). Facilitating health behaviour change and its maintenance: interventions based on self-determination theory. Eur J Health Psychol.

[16] Ryan RM, Deci EL (2017). Self-Determination Theory: Basic Psychological Needs in Motivation, Development, and Wellness.

[17] Williams GC, McGregor HA, Sharp D, Levesque C, Kouides RW, Ryan RM, Deci EL (2006). Testing a self-determination theory intervention for motivating tobacco cessation: supporting autonomy and competence in a clinical trial. Health Psychol.

[18] Sheeran P, Wright CE, Avishai A, Villegas ME, Lindemans JW, Klein WM, Rothman AJ, Miles E, Ntoumanis N (2020). Self-determination theory interventions for health behavior change: meta-analysis and meta-analytic structural equation modeling of randomized controlled trials. J Consult Clin Psychol.

[19] Sheeran P, Wright CE, Avishai A, Villegas ME, Rothman AJ, Klein WM (2021). Does increasing autonomous motivation or perceived competence lead to health behavior change? A meta-analysis. Health Psychol.

[20] Webber KH, Tate DF, Ward DS, Bowling JM (2010). Motivation and its relationship to adherence to self-monitoring and weight loss in a 16-week Internet behavioral weight loss intervention. J Nutr Educ Behav.

[21] Patel ML, Wakayama LN, Bennett GG (2021). Self-monitoring via digital health in weight loss interventions: a systematic review among adults with overweight or obesity. Obesity (Silver Spring).

[22] Payne JE, Turk MT, Kalarchian MA, Pellegrini CA (2018). Defining adherence to dietary self-monitoring using a mobile app: a narrative review. J Acad Nutr Diet.

[23] Nagin DS, Jones BL, Passos VL, Tremblay RE (2018). Group-based multi-trajectory modeling. Stat Methods Med Res.

[24] Nagin DS, Odgers CL (2010). Group-based trajectory modeling in clinical research. Annu Rev Clin Psychol.

[25] Yang Q, Hatch D, Crowley MJ, Lewinski AA, Vaughn J, Steinberg D, Vorderstrasse A, Jiang M, Shaw RJ (2020). Digital phenotyping self-monitoring behaviors for individuals with type 2 diabetes mellitus: observational study using latent class growth analysis. JMIR Mhealth Uhealth.

[26] Hall KL, Nahum-Shani I, August GJ, Patrick ME, Murphy SA, Almirall D, Sloboda Z, Petras H, Robertson E, Hingson R (2019). Adaptive intervention designs in substance use prevention. Prevention of Substance Use.

[27] Golbus JR, Dempsey W, Jackson EA, Nallamothu BK, Klasnja P (2021). Microrandomized trial design for evaluating just-in-time adaptive interventions through mobile health technologies for cardiovascular disease. Circ Cardiovasc Qual Outcomes.

[28] Collins LM, Kugler KC (2018). Optimization of Behavioral, Biobehavioral, and Biomedical Interventions: The Multiphase Optimization Strategy (MOST).

[29] Du Y, Wang J, Li S, Dennis B, Meireles C, Siddiqui N, Patel D, Gelfond J, Li C, Faruqui S, Alaeddini A, Drel V, Tumova J, Ye H, Montellano R, Armaiz-Pena G, Sharma K (2022). A technology assisted precision ketogenic diet intervention for cardio-renal-metabolic health in overweight or obese adults: protocol for a randomized controlled trial. Contemp Clin Trials.

[30] (2003). Adherence to long-term therapies: evidence for action. World Health Organization.

[31] Turner-McGrievy GM, Dunn CG, Wilcox S, Boutté AK, Hutto B, Hoover A, Muth E (2019). Defining adherence to mobile dietary self-monitoring and assessing tracking over time: tracking at least two eating occasions per day is best marker of adherence within two different mobile health randomized weight loss interventions. J Acad Nutr Diet.

[32] Schumacher LM, Martinelli MK, Convertino AD, Forman EM, Butryn ML (2021). Weight-related information avoidance prospectively predicts poorer self-monitoring and engagement in a behavioral weight loss intervention. Ann Behav Med.

[33] Thomson JL, Landry AS, Zoellner JM, Tudor-Locke C, Webster M, Connell C, Yadrick K (2012). Several steps/day indicators predict changes in anthropometric outcomes: HUB City Steps. BMC Public Health.

[34] Levesque CS, Williams GC, Elliot D, Pickering MA, Bodenhamer B, Finley PJ (2007). Validating the theoretical structure of the Treatment Self-Regulation Questionnaire (TSRQ) across three different health behaviors. Health Educ Res.

[35] Williams GC, Niemiec CP, Patrick H, Ryan RM, Deci EL (2009). The importance of supporting autonomy and perceived competence in facilitating long-term tobacco abstinence. Ann Behav Med.

[36] Xu R, Bannor R, Cardel MI, Foster GD, Pagoto S (2023). How much food tracking during a digital weight-management program is enough to produce clinically significant weight loss?. Obesity (Silver Spring).

[37] Wilson J, Heinsch M, Betts D, Booth D, Kay-Lambkin F (2021). Barriers and facilitators to the use of e-health by older adults: a scoping review. BMC Public Health.

[38] Venditti EM, Wylie-Rosett J, Delahanty LM, Mele L, Hoskin MA, Edelstein SL (2014). Short and long-term lifestyle coaching approaches used to address diverse participant barriers to weight loss and physical activity adherence. Int J Behav Nutr Phys Act.

[39] Carpenter CA, Eastman A, Ross KM (2022). Consistency with and disengagement from self-monitoring of weight, dietary intake, and physical activity in a technology-based weight loss program: exploratory study. JMIR Form Res.

[40] Fausset CB, Mitzner TL, Price CE, Jones BD, Fain BW, Rogers WA (2013). Older adults' use of and attitudes toward activity monitoring technologies. Proc Hum Factors Ergon Soc Annu Meet.

[41] Schwarzer R (2016). Health Action Process Approach (HAPA) as a theoretical framework to understand behavior change. Act Psic.

[42] Sniehotta FF, Scholz U, Schwarzer R (2005). Bridging the intention–behaviour gap: planning, self-efficacy, and action control in the adoption and maintenance of physical exercise. Psychol Health.

[43] Trouilloud D, Regnier J (2013). Therapeutic education among adults with type 2 diabetes: effects of a three-day intervention on perceived competence, self-management behaviours and glycaemic control. Glob Health Promot.

[44] Byrne S, Barry D, Petry NM (2012). Predictors of weight loss success. Exercise vs. dietary self-efficacy and treatment attendance. Appetite.

[45] Krukowski RA, Harvey J, Borden J, Stansbury ML, West DS (2022). Expert opinions on reducing dietary self-monitoring burden and maintaining efficacy in weight loss programs: a Delphi study. Obes Sci Pract.

[46] Attig C, Franke T (2020). Abandonment of personal quantification: a review and empirical study investigating reasons for wearable activity tracking attrition. Comput Human Behav.

[47] Hufford MR, Shiffman S, Paty J, Stone AA, Fahrenberg J, Myrtek M (2001). Ecological Momentary Assessment: real-world, real-time measurement of patient experience. Progress in Ambulatory Assessment: Computer-Assisted Psychological and Psychophysiological Methods in Monitoring and Field Studies.

