# Supplementary material for: Understanding Heterogeneity in Individual Responses to Digital Lifestyle Intervention Through Self-Monitoring Adherence Trajectories in Adults With Overweight or Obesity: Secondary Analysis of a 6-Month Randomized Controlled Trial
Source: J Med Internet Res. 2024 Mar 20;26:e53294. doi: 10.2196/53294 (PMC10993111; doi:10.2196/53294)
Supplement: Multimedia Appendix 1 [file jmir_v26i1e53294_app1.docx]

**Multimedia Appendix 1. Group-based multitrajectory model procedures.**

**GBMM Procedures**

In this study, we used package STATA PROC TRAJ to fit GBMM using censored normal distribution to model SM trajectories with a polynomial function of time.

For each individual, we calculated adherence to each self-monitoring (SM) target over 6 months at two-week interval.

Table S1. Data structure for each participant

| Participant | Variables | Week 1-2 | Week 3-4 | … | Week 23-24 |
| --- | --- | --- | --- | --- | --- |
| 1 | Diet SM | 7 | 9 | … | 2 |
|  | Activity SM | 14 | 13 | … | 10 |
|  | Weight SM | 10 | 8 | … | 5 |

Since the number and shape of distinct SM trajectories is unknown, we used a 2-step approach to identify the best-fitting GBMM model as laid out in Nagin et al [23].

***STEP 1:*** We first estimated group-based trajectory models with 2-5 number of groups and modeled each SM adherence outcome as a linear or quadratic function of time separately. This helped us to identify the types of distinctive trajectories that were important to be represented in the GBMM model.

***STEP 2:*** we estimated GBMM models for 3 SM adherence indices jointly with 2-4 groups. Within each model, linear and quadratic terms were tested to model joint SM trajectory as a function of time.

Table S2. Comparison of GBMM with 2-4 subgroups.

|  |  |  |  | Odds of Correct Classification | | | | % group membership | | | |
| --- | --- | --- | --- | --- | --- | --- | --- | --- | --- | --- | --- |
| Number of Groups | BIC for Total N | BIC (Sample Adjusted) | Log Bayes Factor | 1 | 2 | 3 | 4 | 1 | 2 | 3 | 4 |
| 2 | -1370.54 | -1341.23 |  | 61.02 | 695.50 |  |  | 21 (42%) | 29 (58%) |  |  |
| 3 | -1226.77 | -1184.64 | 313.18 | 3995.93 | 1363.81 | 204.74 |  | 10 (20%) | 21 (42%) | 19 (38%) |  |
| 4 | -1187.24 | -1132.28 | 104.72 | 891 | 353.05 | 526.70 | 176 | 5 (10%) | 11 (22%) | 16 (32%) | 18 (36%) |

Figure S1. Visualize multiple SM adherence with GBMM: two groups (final model)

Figure S2. Visualize multiple SM adherence with GBMM: three groups

Figure S3. Visualize multiple SM adherence with GBMM: four groups
